# Cochlear implant in patients with autistic spectrum disorder—a systematic review

**DOI:** 10.1016/j.bjorl.2020.11.020

**Published:** 2021-01-02

**Authors:** Flávia da Silva Tavares, Yaná Jinkings Azevedo, Luísa da Matta Machado Fernandes, Alice Takeuti, Larissa Vilela Pereira, Alelluia Lima Losno Ledesma, Fayez Bahmad

**Affiliations:** a Universidade de Brasília (UnB), Programa de Pós-Graduação em Ciências da Saúde, Brasília, DF, Brazil; b Instituto René Rachou – Fiocruz Minas, FESF-TECH Bahia e Grupo de pesquisa em Políticas de Saúde e Proteção Social, Belo Horizonte, MG, Brazil; c Universidade de Brasília (UnB), Faculdade de Ciências de Saúde, Programa de Pós-Graduação em Ciências da Saúde, Brasília, DF, Brazil

**Keywords:** Autism spectrum disorder, Autistic disorder, Cochlear implant, Cochlear implantations, Systematic review auditory

## Abstract

**Introduction:**

In cases of autism spectrum disorders with severe to profound hearing loss, cochlear implant is a therapeutic option.

**Objective:**

To identify evidence in the scientific literature that the cochlear implant brings benefits to people with autism spectrum disorders with associated hearing loss.

**Methods:**

Systematic review of the literature based on the criteria recommended by PRISMA. The population, intervention, comparison, outcomes, study design, PICOS strategy, was used to define the eligibility criteria. The studies that met the inclusion criteria for this second stage were included in a qualitative synthesis. Each type of study was analyzed according to the Joanna Briggs Institute's risk of bias assessment through the critical checklist for cohort studies, prevalence studies and critical criteria and case reports.

**Results:**

Four hundred and eighty-four articles were found in eight databases and 100 in the gray literature, mentioning the relationship between cochlear implants in patients with autism spectrum disorder and hearing loss. Twelve articles were read in full and 7 were selected for qualitative analysis in this systematic review. All seven articles were analyzed on the critical evaluation checklist. Four articles had a low risk of bias and three articles had a moderate risk of bias. In this study, were included 66 patients with autism spectrum disorder and hearing loss who received cochlear implant.

**Conclusion:**

This systematic review indicates that a cochlear implant can bring benefits to autism spectrum disorder patients with associated deafness.

## Introduction

There is an increase in the number of patients with autism spectrum disorders (ASDs) who constitute a group of developmental disabilities characterized by social interaction and communication impairments. ASDs also present restricted, repetitive, and stereotyped patterns of behavior. Symptoms typically are apparent before age three years.^1^ A study conducted in the United States in 2012 showed that the prevalence of 14.5 per 1000 (one in 69). The estimated prevalence was significantly higher among boys (23.4 per 1.000) than among girls (5.2 per 1000).^2^ There is still no official data on the prevalence of this health condition in Brazil. Individuals with autism differ markedly in the number and severity of symptoms displayed.^3^ Typical signs of autism include but are not limited to speech and language delay, regression of developmental milestones at 18–24 months of age, avoidance of eye contact, tactile defensiveness, and engagement in repetitive and self-stimulating behaviors. Approximately 80% of children with autism have some degree of cognitive impairment.^4^

In addition, some people with ASD may have associated hearing loss. Beers et al.^5^ carried out a systematic review (SR) and found that the prevalence of hearing loss among individuals with ASD is controversial. Studies aim to find a higher incidence of hearing loss among people with ASD than in the general population.^6^, ^7^, ^8^ The authors also warned of the difficulty in generalizing the prevalence found, considering the studied sample’s limitations. A clear relationship was not found between the severity of autistic behavior and the degree of hearing loss.^8^ Gravel et al.^9^ found no evidence of differences in the peripheral auditory system between children with ASD and their typically developing peers.

In cases where ASD and hearing impairment co-exists, diagnosis of one condition often leads to a delay in diagnosing the other.^8^, ^10^ The diagnosis of hearing loss may have obscured recognition of autistic behaviors added up five years.^10^ It is recommended that children receive a complete audiological assessment when ASD is suspected. That way, the peripheral hearing loss can be diagnosed early and managed as part of the child's habilitation and education program.^5^, ^7^, ^10^ The cochlear implant (CI) is a therapeutic option for cases of ASD with associated deafness. CI is the treatment of choice for children with severe to profound sensorineural hearing loss.^11^ This implant is a high-tech electronic device developed to perform the function of cochlear hair cells that are damaged or missing, intending to provide electrical stimulation of the remaining auditory nerve fibers.^12^

This SR aims to identify evidence in the scientific literature that the CI favors auditory development, language, and social interaction in people with ASD with associated severe and/or profound hearing loss.

## Methods

This SR's search strategy followed the criteria recommended by the Preferred reporting items for systematic reviews and meta-analyses – PRISMA.^13^ The protocol was registered on April 27^th^, 2020, at the International prospective register of systematic reviews – PROSPERO (https://www.crd.york.ac.uk/PROSPERO/) registration number CRD4202015045.

### Search strategy

The search strategy was performed in English, and the databases used were: PubMed, Cochrane, Lilacs, Livivo, Medline, Science Direct, Scopus, and Web of Science. The gray literature was consulted through the Google Scholar database. There was no restriction on the period or language of publication.

The keywords of the search strategy to identify articles published until September 2019 were described and combination as follows: “autism spectrum disorder” OR “autistic disorder” OR “autism” OR “autistic spectrum” (AND) “cochlear implant” OR “cochlear implants” OR “cochlear implantation” OR “cochlear implantations”. This same search strategy was used in all databases and gray literature.

After the search, each database's references were exported to the EndNote X9 program (https://endnote.com), and then these same references were exported from EndNote X98 to the Rayyan QCRI program (https://rayyan.qcri.org/welcome). The purpose of these two programs was to record all duplicate articles found in the scientific literature, promoting greater reliability in selecting articles and proceeding to the eligibility stage.

### Eligibility criteria

The population, intervention, comparison, outcomes, study design(s) (PICOS)^13^ strategy was used to define the eligibility criteria. The inclusion criteria was: 1) Population: patients with ASD and severe and/or profound hearing loss who used CI; 2) Intervention: received a CI at any age and be diagnosed with ASD at any age; 3) Comparison: development of hearing, language, and social interaction skills before and after CI surgery in each individual as well as comparing the development of these skills in patients who have only ASD and those with other associated disabilities; 4) Outcomes: evaluation of behavioral changes or communication skills after using CI13; 5) Study design: prospective clinical cohort, clinical cases, and case reports.

The exclusion criteria were: (1) Articles with patients not considered for a cochlear implant, (2) Studies using animals and in vitro, and (3) Studies with a lack of postoperative data.

All studies were analyzed for eligibility in the screening phases based on the inclusion and exclusion criteria. In the first phase, all the studies were selected based on two reviewers’ titles and abstracts analysis. There was no disagreement among the reviewers in this phase, ruling out the need to consult the third reviewer. In the case of summary abstention, but with a relevant title, the study was included in the second phase.

In the second phase, the same two reviewers read each selected article’s full text using the same inclusion and exclusion criteria, but adding the exclusion justification for each discarded study. The studies that met the inclusion criteria in this second step were included in a qualitative synthesis. Each type of study was analyzed according to the bias risk of bias assessment of the Joanna Briggs Institute (JBI).^14^

### Qualitative synthesis

The instruments used for the risk of bias assessment were the validated JBI critical appraisal checklists for each study design: cohort studies, studies reporting prevalence data, and case report. In the JBI critical assessment checklist, each question must be answered through four options: yes (Y), no (N), unclear (U), and not applicable (NA). The bias risk percentage calculation is done by the amount of “Y” selected in the checklist. When “NA” was selected, the question was not considered in the calculation, according to the Joanna Briggs Institute (JBI).^14^ Up to 49% is considered a high risk of bias, 50%–70% is moderate, and above 70%, there is a low risk of bias.

In this phase, the same two reviewers applied the bias risk assessment of the JBI independently. There was no disagreement between them, ruling out the need to consult the third reviewer.

## Results

**Figure 1:**
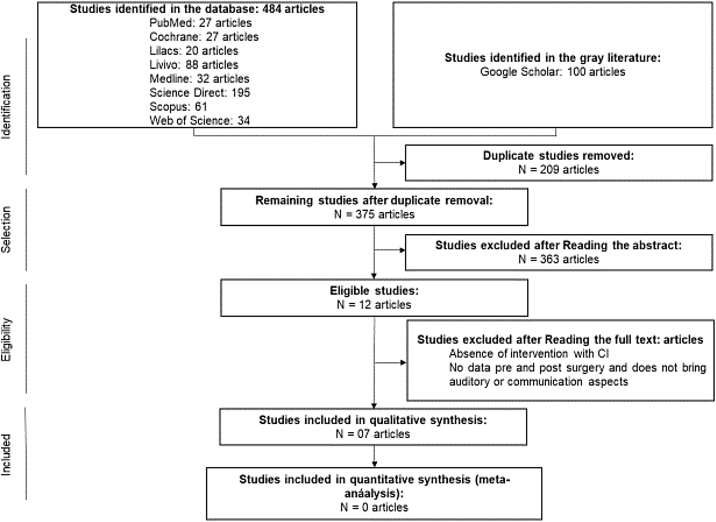
Diagram of the identification and selection of articles adapted from PRISMA.

The first phase of this SR found 484 articles in eight databases and 100 in the gray literature. After eliminating 209 duplicate studies, 375 were selected by reviewers to read titles and abstracts. Of these, 363 articles were excluded by the established exclusion criteria, and twelve articles were included in the second stage, which consisted of reading the full manuscript. Four articles were excluded in this stage for the following reasons: two articles^15^, ^16^ showed the absence of a subject with ASD + CI; two^17^, ^18^ did not present pre- and postoperative data of the subjects, present the mother’s perception, and do not show auditory or communication aspects. Seven studies^11^, ^19^, ^20^, ^21^, ^22^, ^23^, ^24^ were selected for qualitative analysis in the present SR (Table 1). No studies were found by performing a manual search of the references of the articles. The whole article selection process is described in Fig. 1, which shows the flow PRISMA diagram for inclusion.

**Table 1:** Selected studies following the inclusion and exclusion criteria established in the SR.

	Title	Author	Location	Year of publication	Study design	Total N
1	Measuring progress in children with autism spectrum disorder who have cochlear implants	Donaldson et al.^19^	Michigan—USA	2004	Cohort studies	7
2	Cochlear implant candidacy in children with autism	Hayman and Franck^20^	Philadelphia, Pennsylvania—USA	2005	Case reports	3
3	Children with cochlear implants and autism—challenges and outcomes: the experience of the National Cochlear implant program, Ireland	Robertson^11^	Dublin—Ireland	2013	Case reports	10
4	Cochlear implantation in children with autism spectrum disorder	Eshraghi et al.^21^	Miami—USA	2015	Cohort studies	15
5	Receptive speech in early implanted children later diagnosed with autism	Mikic et al.^22^	Belgrade—Serbia	2016	Cohort studies	14
6	Compliance with cochlear implantation in children subsequently diagnosed with autism spectrum disorder	Valero et al.^23^	Manchester, UK	2016	Prevalence studies	22
7	Cochlear implantation in autistic children with profound sensorineural hearing loss	Lachowska et al.^24^	Warsaw—Polônia	2018	Prevalence studies	6

All seven articles were analyzed according to the JBI^14^ according to each type of study: JBI Critical Appraisal Checklist for Cohort Studies (Table 2), JBI Critical Appraisal Checklist for Studies Reporting Prevalence Data (Table 3), and JBI Critical Appraisal Checklist for Case Report (Table 4). Four articles^11^, ^20^, ^21^, ^22^ showed a low risk of bias, and three article^19^, ^23^, ^24^ showed a moderated risk of bias.

**Table 2:** JBI critical appraisal checklist for cohort studies.

	Donaldson et al.^19^	Eshraghi et al.^21^	Mikic et al.^22^
Were the two groups similar and recruited from the same population?	Y	Y	Y
Were the exposures measured similarly to assign people to both exposed and unexposed groups?	U	Y	Y
Was the exposure measured in a valid and reliable way?	Y	Y	Y
Were confounding factors identified?	N	Y	Y
Were strategies to deal with confounding factors stated?	NA	Y	U
Were the groups/participants free of the outcome at the start of the study (or at the moment of exposure)?	U	Y	Y
Were the outcomes measured in a valid and reliable way?	Y	Y	Y
Was the follow-up time reported and sufficient to be long enough for outcomes to occur?	Y	Y	Y
Was follow up complete, and if not, were the reasons to loss to follow up described and explored?	Y	Y	Y
Were strategies to address incomplete follow up utilized?	U	Y	U
Was an appropriate statistical analysis used?	U	Y	Y
Bias risk (%)	50%	100%	81.81%

Y, yes; N, no; U, unclear; NA, not applicable.

**Table 3:** JBI critical appraisal checklist for studies reporting prevalence data.

	Valero et al.^23^	Lachowska et al.^24^
Was the sample frame appropriate to address the target population?	Y	Y
Were study participants sampled in an appropriate way?	Y	Y
Was the sample size adequate?	U	U
Were the study subjects and the setting described in detail?	U	U
Was the data analysis conducted with sufficient coverage of the identified sample?	U	U
Were valid methods used for the identification of the condition?	Y	Y
Was the condition measured in a standard, reliable way for all participants?	Y	Y
Was there an appropriate statistical analysis?	U	U
Was the response rate adequate, and if not, was the low response rate managed appropriately?	Y	Y
Bias risk (%)	55%	55%

Y, yes; U, unclear.

**Table 4:** JBI critical appraisal checklist for case report.

	Hayman and Franck^20^	Robertson^11^
Were patient’s demographic characteristics clearly described?	Y	Y
Was the patient’s history clearly described and presented as a timeline?	Y	Y
Was the current clinical condition of the patient on presentation clearly, described?	Y	Y
Were diagnostic tests or assessment methods and the results clearly, described?	Y	Y
Was the intervention(s) or treatment procedure(s) clearly described?	Y	Y
Was the post-intervention clinical condition clearly described?	Y	Y
Were adverse events (harms) or unanticipated events identified and described?	Y	Y
Does the case report provide takeaway lessons?	Y	Y
Bias risk (%)	100%	100%

Y, yes.

In the seven studies selected for qualitative analysis, 67 individuals with ASD and hearing loss were described. Of these, 66 received CI (for one subject, CI was contraindicated). Forty-one subjects (62%) were diagnosed with ASD after the CI, 7 (11%) before the CI, and for 18 subjects (27%), the authors did not say whether the diagnosis of ASD was before or after the CI. Not all subjects had the same characteristics within the ASD. Two (2) individuals were ASD without intellectual and linguistic deficits (formerly known as Asperger syndrome), five (5) with PDD-NOS (Pervasive Developmental Disorder – Not Otherwise Specified), and twenty-four (24) with autism. When analyzing the research subjects’ description with ASD, it was observed that 31 of them (46.3%) had some other associated disability, and 35 (53.7%) did not present reports of other associated disabilities.

The characteristics of patients with ASD and hearing loss who received a cochlear implant, without other associated disabilities, are shown in Table 5. Table 6 presents the results of patients with ASD and hearing loss who received a CI and have other associated disabilities.

**Table 5:** Demographic data and implant details for patients with autistic spectrum disorder and hearing loss with the cochlear implant without other associated disabilities.

	Author	Sex	Age at implant	Associated disabilities	ADS type	Year of implantation	Test	Results
1	Donaldson et al.^19^	F	9-years	U	PDD-NOS—3-years before – preimplant, 6-years-old	Between 1998 and 2003	MacArthur Communication Development Inventory, EVT, PPVT-III, MAIS/IT-MAIS, GASP-W, GASP-S, Parental ratings	Demonstrated an increase in their comprehension of spoken words after implantation
2	Donaldson et al.^19^	M	4-years	U	PDD-NOS—Pervasive developmental disorder, 3-years post-implant 7-years-old	Between 1998 and 2003	MacArthur Communication Development Inventory, EVT, PPVT-III, MAIS/IT-MAIS, GASP-W, GASP-S, Parental ratings	Demonstrated an increase in their comprehension of spoken words after implantation. Showed improvement in the auditory comprehension test score, going from a standard below 40 in the preoperative period to 72 stitches 5 years after surgery Demonstrated strong improvements in the Expressive Vocabulary Test, going from 54 points in the 24a month after implant until 81 60 months after implant Achieved a score of 100% correct on both words and sentences two years after implantation
3	Donaldson et al.^19^	M	7-years	U	Autism, 2 years before – preimplant, 5-years-old	Between 1998 and 2003	MacArthur Communication Development Inventory, EVT, PPVT-III, MAIS/IT-MAIS, GASP-W, GASP-S, Parental ratings	Communication presented with other people through sign language and gestures
4	Donaldson et al.^19^	M	3-years	U	Autism, 1-year post-implant—4-years-old	Between 1998 and 2003	MacArthur Communication Development Inventory, EVT, PPVT-III, MAIS/IT-MAIS, GASP-W, GASP-S, Parental ratings	Demonstrated an increase in their comprehension of spoken words after implantation
5	Donaldson et al.^19^	M	8-years	U	Autism, 3-years before—preimplant, 5-years-old	Between 1998 and 2003	MacArthur Communication Development Inventory, EVT, PPVT-III, MAIS/IT-MAIS, GASP-W, GASP-S, Parental ratings	Demonstrated an increase in their comprehension of spoken words after implantation
6	Donaldson et al.^19^	M	3-years	U	Autism, 2 years post-implant—5-years-old	Between 1998 and 2003	MacArthur Communication Development Inventory, EVT, PPVT-III, MAIS/IT-MAIS, GASP-W, GASP-S, Parental ratings	Demonstrated no comprehension of spoken words preoperatively or at the 12 months post-activation interval.
7	Donaldson et al.^19^	M	3-years	U	Autism, 1-year before—preimplant, 2-years-old	Between 1998 and 2003	MacArthur Communication Development Inventory, EVT, PPVT-III, MAIS/IT-MAIS, GASP-W, GASP-S, Parental ratings	Demonstrated an increase in their comprehension of spoken words after implantation
8	Hayman and Franck^20^	M	11.3 years	None	Autism, preimplant	U	Early Speech Perception Test, MLNT	Have a total communication class with other children with cochlear implants. He is reading on almost a 3rd-grade level, and educators believe it will continue to improve.
9	Robertson^11^	U	2.1 years	None	Autism, 8 months post-implant—2,9-year-old	2005	U	PECS (Picture Exchange Communication System)
10	Robertson^11^	U	2.6 years	None	Autism, 7-years post-implant—9-years-old	2005	U	ISL (Irish Sign Language)
11	Robertson^11^	U	2-years	None	Autism, 2.1-years post-implant—4.1-years-old	2007	U	Spoken language Understands common phrases. Speech intelligible to familiar listeners
12	Eshraghi et al.^21^	U	4 (1) and 10 (2) years	None	Autism not informed if pre- or post-implant	Between 1992 and 2011	Speech perception and speech expression evaluation. ABR, ESP test, MLNT, PBK test Parental survey: 39 questions evaluating the subjective impression of CI benefits	Speech Perception: changed of “No awareness of environment” for “Identification/recognition of simple phrases (2 words) and commands”. Speech Expression: changed of “No vocalization” for “Simple Phrases and Commands (Where is X, let us go, etc.)”
13	Eshraghi et al.^21^	U	1.5 (1) and 6 (2) years	None	AutismNot informed if pre- or post-implant	Between 1992 and 2011	Speech perception and speech expression evaluation. ABR, ESP test, MLNT, PBK test Parental survey: 39 questions evaluating the subjective impression of CI benefits	Speech Perception: changed of “No awareness of environment” for “Understands conversations”. Speech Expression: changed of “No vocalization” for “Simple Phrases and Commands (Where is X, let's go, etc.)”
14	Eshraghi et al.^21^	U	3-years	None	AutismNot informed if pre- or post-implant	Between 1992 and 2011	Speech perception and speech expression evaluation. ABR, ESP test, MLNT, PBK test Parental survey: 39 questions evaluating the subjective impression of CI benefits	Speech Perception: changed of “No awareness of environment” for “Understands conversations”. Speech Expression: changed of “Some vocalization (consonants, vowels, nasal sounds)” for “Simple Phrases and Commands (Where is X, let's go, etc.)”
15	Eshraghi et al.^21^	U	4 (1) and 15 (2) years	None	Autism not informed if pre- or post-implant	Between 1992 and 2011	Speech perception and speech expression evaluation. ABR, ESP test, MLNT, PBK test Parental survey: 39 questions evaluating the subjective impression of CI benefits	Speech Perception: changed of “Identification/recognition of words” for “Understands conversations”. Speech Expression: changed of “No vocalization” for “Able to produce sentences”.
16	Eshraghi et al.^21^	U	1.67 years	None	PDD-NOS (Pervasive developmental disorder—not otherwise specified) not informed if pre- or post-implant	Between 1992 and 2011	Speech perception and speech expression evaluation ABR, ESP test, MLNT, PBK test Parental survey: 39questions evaluating the subjective impression of CI benefits	Speech Perception: changed of “No awareness of environment” for “Understands conversations”. Speech Expression: changed of “No vocalization” for “Able to produce sentences”
17	Eshraghi et al.^21^	U	4-years	None	PDD-NOS (Pervasive developmental disorder—not otherwise specified) not informed if pre- or post-implant	Between 1992 and 2011	Speech perception and speech expression evaluation ABR, ESP test, MLNT, PBK test Parental survey: 39questions evaluating the subjective impression of CI benefits	Speech Perception: changed of “Identification/ recognition of words” for “Identification/ recognition of simple phrases (2-words) and commands”. Speech Expression: unchanged. Kept “Some vocalization (consonants, vowels, nasal sounds)”
18	Eshraghi et al.^21^	U	2-years	None	PDD-NOS (Pervasive developmental disorder—not otherwise specified) not informed if pre- or post-implant	Between 1992 and 2011	Speech perception and speech expression evaluation ABR, ESP test, MLNT, PBK test Parental survey: 39questions evaluating the subjective impression of CI benefits	Speech Perception: changed of “No awareness of environment” for “Identification/ recognition of simple phrases (2-words) and commands”. Speech Expression: changed of “No vocalization” for “Simple Phrases and Commands (Where is X, let's go, etc.)”
19	Eshraghi et al.^21^	U	1.5 (1) and 2 (2) years	None	Autism not informed if pre- or post-implant	Between 1992 and 2011	Speech perception and speech expression evaluation ABR, ESP test, MLNT, PBK test Parental survey: 39questions evaluating the subjective impression of CI benefits	Speech Perception: Understands conversations; Speech Expression: Able to produce sentences
20	Mikic et al.^22^	M	0.9-year	U	Autism not informed if pre- or post-implant	Between 2008 and 2009	Categories of Auditory Performance (CAP); Speech Intelligibility Rating (SIR)	Auditory processing developed slowly. Could identify environmental sounds or discriminate speech sounds, with very little progress up to six years old, despite extensive speech and language therapy.
21	Mikic et al.^22^	M	1-year	U	Autism not informed if pre- or post-implant	Between 2008 and 2009	Categories of Auditory Performance (CAP); Speech Intelligibility Rating (SIR)	Auditory processing developed slowly. Could identify environmental sounds or discriminate speech sounds, with very little progress up to six years old, despite extensive speech and language therapy.
22	Mikic et al.^22^	F	0.8-year	U	Autism not informed if pre- or post-implant	Between 2008 and 2009	Categories of Auditory Performance (CAP); Speech Intelligibility Rating (SIR)	Maintained the same pre- and post-IC characteristics Auditory processing developed slowly. Could identify environmental sounds or discriminate speech sounds, no progress up to six years old, despite extensive speech and language therapy.
23	Mikic et al.^22^	M	1-year	U	Autism not informed if pre- or post-implant	Between 2008 and 2009	Categories of Auditory Performance (CAP); Speech Intelligibility Rating (SIR)	Auditory processing developed slowly. Could identify environmental sounds or discriminate speech sounds, with very little progress up to six years old, despite extensive speech and language therapy.
24	Valero et al.^23^	F	2.10 years	None	Autistic spectrum, post-implant	Between 1989 and 2015	Speech perception and expression categories (adapted from Yeargin-Alisopp et al. 2003)	Non-user speech perception, No vocalization, Non-user the CI, Sign Communication
25	Valero et al.^23^	M	1.10 years	None	Autistic spectrum, post-implant	Between 1989 and 2015	Speech perception and expression categories (adapted from Yeargin-Alisopp et al. 2003)	Identification/recognition of words Some vocalization (consonants, vowels, nasal sounds) User the CI Some vocalization and PECS (Picture Exchange Communication System) Communication
26	Valero et al.^23^	M	3.8 years	None	Autistic spectrum, post-implant	Between 1989 and 2015	Speech perception and expression categories (adapted from Yeargin-Alisopp et al. 2003)	Understands conversations, Able to produce sentences, User the CI, Oral Communication
27	Valero et al.^23^	M	2.4 years	None	Autistic spectrum, post-implant	Between 1989 and 2015	Speech perception and expression categories (adapted from Yeargin-Alisopp et al. 2003)	Identification/recognition of words Words only User the CI Words Communication
28	Valero et al.^23^	M	1-year	None	Autistic spectrum, post-implant	Between 1989 and 2015	Speech perception and expression categories (adapted from Yeargin-Alisopp et al. 2003)	Identification/recognition of simple phrases (two words) and commands, Words only, User the CI, Word, sing and PECS (Picture Exchange Communication System) Communication
29	Valero et al.^23^	M	1.10 years	None	PDD-NOS (Pervasive developmental disorder—not otherwise specified), post-implant	Between 1989 and 2015	Speech perception and expression categories (adapted from Yeargin-Alisopp et al. 2003)	Non-user speech perception, No vocalization, Non-user the CI, None Communication
30	Lachowska et al.^24^	M	1.9 years	None	Autism post-implant	U	Medical history, Reaction to music and sound, Ling's six sounds test, Onomatopoeic word test, Reaction to spoken child’s name, Response to requests, the questionnaire given to parents, Sound processor fitting sessions and data	Reaction to music: No, Ling's 6 sounds teste: No, Reaction to spoken name: No, Response to spoken request: No, Response to name: No, Response to environmental sounds: No, Behavior changes: None, Better family interactions: Yes
31	Lachowska et al.^24^	M	2.5 years	None	Autism post-implant	U	Medical history, Reaction to music and sound, Ling's six sounds test, Onomatopoeic word test, Reaction to spoken child’s name, Response to requests, the questionnaire given to parents, Sound processor fitting sessions and data	Reaction to music: Yes (only to drum), Ling's 6 sounds teste: No, Reaction to spoken name: No, Response to spoken request: No, Response to name: No, Response to environmental sounds: No, Behavior changes: None, Better family interactions: Yes
32	Lachowska et al.^24^	M	1.9 years	None	Autism post-implant	U	Medical history, Reaction to music and sound, Ling's six sounds test, Onomatopoeic word test, Reaction to spoken child’s name, Response to requests, the questionnaire given to parents, Sound processor fitting sessions and data	Reaction to music: Yes (only to drum), Ling's 6 sounds teste: No, Reaction to spoken name: No, Response to spoken request: No, Response to name: No, Response to environmental sounds: No, Behavior changes: Reduced anxiety, Better family interactions: Yes
33	Lachowska et al.^24^	M	1.9 years	None	Autism post-implant	U	Medical history, Reaction to music and sound, Ling's six sounds test, Onomatopoeic word test, Reaction to spoken child’s name, Response to requests, the questionnaire given to parents, Sound processor fitting sessions and data	Reaction to music: Yes (only to flute and drum), Ling's 6 sounds teste: No, Reaction to spoken name: No, Response to spoken request: No, Response to name: Yes, Response to environmental sounds: Yes, Behavior changes: Reduced anxiety, Better family interactions: Yes
34	Lachowska et al.^24^	M	1.3 years	None	Autism post-implant	U	Medical history, Reaction to music and sound, Ling's six sounds test, Onomatopoeic word test, Reaction to spoken child’s name, Response to requests, the questionnaire given to parents, Sound processor fitting sessions and data	Reaction to music: Yes, Ling's 6 sounds teste: Yes, Reaction to spoken name: Yes, Response to spoken request: Yes, Response to name: Yes, Response to environmental sounds: Yes, Behavior changes: Reduced anxiety, Better family interactions: Yes
35	Lachowska et al.^24^	M	2.2 years	None	Autism post-implant	U	Medical history, Reaction to music and sound, Ling's six sounds test, Onomatopoeic word test, Reaction to spoken child’s name, Response to requests, the questionnaire given to parents, Sound processor fitting sessions and data	Reaction to music: Yes, Ling's 6 sounds teste: Yes, Reaction to spoken name: Yes, Response to spoken request: Yes, Response to name: Yes, Response to environmental sounds: Yes, Behavior changes: Reduced anxiety, Better family interactions: Yes

F, female; M, male; U, uninformed.

**Table 6:** Demographic data and implant details for patients with Autistic Spectrum Disorder and Hearing Loss with a cochlear implant with other associated disabilities.

	Author	Sex	Age at implant	Associated disabilities	ADS type	Year of implantation	Test	Results
1	Hayman and Franck^20^	U	1 year	Cortical blindness Globally delayed	PDD-NOS 2.5-years post-implant—3-year-old	U	U	Socially and emotionally responsive, demonstrates better eye contact and orienting, improved listening response, and improved nonverbal communication such as indicating choices, show of affection, and social preferences, receptive language has improved markedly
2	Robertson^11^	U	3.2 years	Developmental delay/mild intellectual disability	Autism 10-months post-implant—3.10 year-old	2004	U	ISL (Irish Sign Language)/spoken language. Understands common phrases. Speech unintelligible
3	Robertson^11^	U	2,4 years	Verbal dyspraxia	Autism 1.2-year post-implant—3.6-year-old)	2005	U	ISL (Irish Sign Language)/spoken language. Understands common phrases. Speech unintelligible
4	Robertson^11^	U	4.4 years	Cerebral Palsy/intellectual disability	Autism 1.1-year post-implant—5.5-year-old)	2005	U	PECS (Picture Exchange Communication System)/LAMH (Language Alternative for the Mentally Handicapped)
5	Robertson^11^	U	3.10 years	Epilepsy, left hemiparesis	Autism 1.0-year post-implant—4.10-year-old)	2007	U	ISL (Irish Sign Language)/spoken language. Understands common phrases. Speech unintelligible
6	Robertson^11^	U	2.3 years	Intellectual disability/visual diffs/multiple medical problems	Autism, 10 months post-implant—3.1-years-old	2009	U	PECS (Picture Exchange Communication System)/LAMH (Language Alternative for the Mentally Handicapped)
7	Robertson^11^	U	13.1 years	Intellectual disability	Autism, 7.5-years before—pre-implant, 5.6-years-old	2010	U	PECS (Picture Exchange Communication System)/LAMH (Language Alternative for the Mentally Handicapped)
8	Robertson^11^	U	7-years	Intellectual disability	Autism, 4-years before—pre-implant, 3-years-old	2011	U	PECS (Picture Exchange Communication System)/LAMH (Language Alternative for the Mentally Handicapped)
9	Eshraghi et al.^21^	U	1.75 years	Rumination, GERD (Gastroesophageal reflux disease), Strabismus	Autism not informed if pre or post-implant	Between 1992 and 2011	Speech perception and speech expression evaluation ABR, ESP test, MLNT, PBK test Parental survey: 39 questions evaluating the subjective impression of CI benefits	Speech Perception: unchanged. Kept Awareness, detection, or localization of sound Speech Expression: unchanged. Kept “Some vocalization (consonants, vowels, nasal sounds)”
10	Eshraghi et al.^21^	U	5.5 years	Gross motor delay, Strabismus	Autism not informed if pre or post-implant	Between 1992 and 2011	Speech perception and speech expression evaluation ABR, ESP test, MLNT, PBK test Parental survey: 39 questions evaluating the subjective impression of CI benefits	Speech Perception: changed of “No awareness of environment” for "Identification/ recognition of words”. Speech Expression: unchanged. Kept “Some vocalization (consonants, vowels, nasal sounds)”
11	Eshraghi et al.^21^	U	4.5 years	Prematurity, encephalopathy, gross motor delay	Autism Not informed if pre or post-implant	Between 1992 and 2011	Speech perception and speech expression evaluation ABR, ESP test, MLNT, PBK test Parental survey: 39 questions evaluating the subjective impression of CI benefits	Speech Perception: changed of “No awareness of environment” for “Awareness, detection or localization of sound”. Speech Expression: unchanged. Kept “Some vocalization (consonants, vowels, nasal sounds)”
12	Eshraghi et al.^21^	U	3.5 years	polymicrogyria, gliosis, developmental delay	Autism Not informed if pre or post-implant	Between 1992 and 2011	Speech perception and speech expression evaluation ABR, ESP test, MLNT, PBK test Parental survey: 39 questions evaluating the subjective impression of CI benefits	Speech Perception: changed of “No awareness of environment” for “Awareness, detection or localization of sound”. Speech Expression: changed of “No vocalization” for “Some vocalization (consonants, vowels, nasal sounds)”
13	Eshraghi et al.^21^	U	2-years	ADHD (Attention Deficit Hyperactivity Disorder)	PDD-NOS 2.5-years post-implant—3-year-old	Between 1992 and 2011	Speech perception and speech expression evaluation ABR, ESP test, MLNT, PBK test Parental survey: 39 questions evaluating the subjective impression of CI benefits	Speech Perception: changed of “Awareness, detection or localization of sound” for “Understands conversations”. Speech Expression: changed of “No vocalization” for “Able to produce sentences”
14	Eshraghi et al.^21^	U	4-years	Ushers Syndrome	Autism Not informed if pre or post-implant	Between 1992 and 2011	Speech perception and speech expression evaluation ABR, ESP test, MLNT, PBK test Parental survey: 39 questions evaluating the subjective impression of CI benefits	Speech Perception: changed of “No awareness of environment” for “Awareness, detection or localization of sound”. Speech Expression: changed of “No vocalization” for “Some vocalization (consonants, vowels, nasal sounds)”
15	Eshraghi et al.^21^	U	3 (1) and 12 (2) years	Meningitis at 18-months	Autism Not informed if pre or post-implant	Between 1992 and 2011	Speech perception and speech expression evaluation ABR, ESP test, MLNT, PBK test Parental survey: 39 questions evaluating the subjective impression of CI benefits	Speech Perception: changed of “No awareness of environment” for “Understands conversations”. Speech Expression: changed of “No vocalization” for “Able to produce sentences”
16	Valero et al.^23^	F	1.8 years	Learning disabilities	Autistic spectrum, post-implant	Between 1989 and 2015	Speech perception and expression categories (adapted from Yeargin-Alisopp et al. 2003)	Non-user speech perception, Some vocalization (consonants, vowels, nasal sounds). Non-user the CI. Some vocalization Communication
17	Valero et al.^23^	M	3.2 years	Prematurity Learning disabilities	Autistic spectrum, post-implant	Between 1989 and 2015	Speech perception and expression categories (adapted from Yeargin-Alisopp et al. 2003)	Identification/recognition of words. Some vocalization (consonants, vowels, nasal sounds) User the CI, PECS (Picture Exchange Communication System) Communication
18	Valero et al.^23^	M	1.8 years	Meningitis Learning disabilities	Autistic spectrum, post-implant	Between 1989 and 2015	Speech perception and expression categories (adapted from Yeargin-Alisopp et al. 2003)	Identification/recognition of simple phrases (two words) and commands; Some vocalization (consonants, vowels, nasal sounds). User the CI. Sign and PECS (Picture Exchange Communication System) Communication
19	Valero et al.^23^	M	3.9 years	Prematurity	Autistic spectrum, post-implant	Between 1989 and 2015	Speech perception and expression categories (adapted from Yeargin-Alisopp et al. 2003)	Non-user speech perception, No vocalization, Non-user the CI, Sign and PECS (Picture Exchange Communication System) Communication
20	Valero et al.^23^	M	1.10 years	Communication disabilities	Autistic spectrum, post-implant	Between 1989 and 2015	Speech perception and expression categories (adapted from Yeargin-Alisopp et al. 2003)	Identification/recognition of words. Words only. User the CISome words, signs, and PECS (Picture Exchange Communication System) Communication
21	Valero et al.^23^	M	5-years	Meningitis, ADHD (Attention-Deficit Hyperkinetic Disorder) Communication disabilities	Autistic spectrum, post-implant	Between 1989 and 2015	Speech perception and expression categories (adapted from Yeargin-Alisopp et al. 2003)	Understands conversations, Able to produce sentences, User the CI, Oral Communication
22	Valero et al.^23^	M	6.11 years	Prematurity, ADHD (Attention-Deficit Hyperkinetic Disorder)	Autistic spectrum, post-implant	Between 1989 and 2015	Speech perception and expression categories (adapted from Yeargin-Alisopp et al. 2003)	Understands conversations, Able to produce sentences, User the CI—unilateral, Oral and sign Communication
23	Valero et al.^23^	M	3-years	Smith-Lemli-Opitz Syndrome Global developmental delay	Autistic spectrum, post-implant	Between 1989 and 2015	Speech perception and expression categories (adapted from Yeargin-Alisopp et al. 2003)	Identification/recognition of simple phrases (two words) and commands, Simple phrases and commands, User the CI—unilateral, Phrases and sign Communication
24	Valero et al.^23^	M	3.1 years	Communication disabilities	Autistic spectrum, post-implant	Between 1989 and 2015	Speech perception and expression categories (adapted from Yeargin-Alisopp et al. 2003)	Identification/recognition of words. Some vocalization (consonants, vowels, nasal sounds) User the CI Some words and sing Communication
25	Valero et al.^23^	M	5.2 years	ADHD (Attention-Deficit Hyperkinetic Disorder) Learning disabilities	Autistic spectrum, post-implant	Between 1989 and 2015	Speech perception and expression categories (adapted from Yeargin-Alisopp et al. 2003)	Identification/recognition of simple phrases (two words) and commands, Words only, User the CI, Word and sing Communication
26	Valero et al.^23^	M	1.3 years	Waardenberg syndrome, Learning disabilities	PDD-NOS post-implant	Between 1989 and 2015	Speech perception and expression categories (adapted from Yeargin-Alisopp et al. 2003)	Non-user speech perception, No vocalization, Non-user the CI, None Communication
27	Valero et al.^23^	M	4.6 years	Learning disabilities	PDD-NOS, post-implant	Between 1989 and 2015	Speech perception and expression categories (adapted from Yeargin-Alisopp et al. 2003)	“Non-user speech perception, No vocalization; Non-user the CI, Sign and PECS (Picture Exchange Communication System) Communication
28	Valero et al.^23^	M	2.8 years	Prematurity Global developmental delay	PDD-NOS, post-implant	Between 1989 and 2015	Speech perception and expression categories (adapted from Yeargin-Alisopp et al. 2003)	Awareness, detection of localization of sound, No vocalization, User the CI, Sign Communication
29	Valero et al.^23^	M	8.2 years	CMV (Cytomegalovirus infection)	Asperger’s disorder, post-implant	Between 1989 and 2015	Speech perception and expression categories (adapted from Yeargin-Alisopp et al. 2003)	Understands conversations, Able to produce sentences, User the CI, Oral and sign Communication
30	Valero et al.^23^	M	2.8 years	Waardenberg syndrome, ADHD (Attention-Deficit Hyperkinetic Disorder)	Asperger’s disorder, post-implant	Between 1989 and 2015	Speech perception and expression categories (adapted from Yeargin-Alisopp et al. 2003)	“Understands conversations, Able to produce sentences, User the CI, Oral Communication”
31	Valero et al.^23^	M	2.10 years	Prematurity, Learning and communication difficulties	Autistic Spectrum, post-implant	Between 1989 and 2015	Speech perception and expression categories (adapted from Yeargin-Alisopp et al. 2003)	Identification/recognition of words. Some vocalization (consonants, vowels, nasal sounds). User the CI. Sing and PECS (Picture Exchange Communication System) Communication

F, female; M, male; U, uninformed.

Among the 35 patients with ASD and hearing loss without other associated disabilities, 6 (17%) did not establish any communication form. However, there was an increase in interaction with family members. Six (17%) did not develop oral communication – however, advanced sign language communication or alternative communication (pictures). Meanwhile, 9 (26%) demonstrated recognition of simple verbal commands and spoke simple sentences; and 14 (40%) developed language and fluent oral communication.

Among the 31 patients who, in addition to ASD and hearing loss, had other disabilities, 15 (48%) did not develop oral communication. Those communicated using sign language or alternative communication by pictures. Twelve patients (39%) demonstrated recognition of single verbal commands and vocalized simple words. Furthermore, four patients (13%) established oral communication in a simplified way after using CI.

All seven articles were analyzed according to the Grades of Recommendation, Assessment, Development, and Evaluation – GRADEpro^25^, ^26^ (Table 7).

**Table 7:** Analyzed according to the Grades of Recommendation, Assessment, Development, and Evaluation – GRADEpro.

Outcome	Nº of studies (Nº of patients)	Study design	Factors that may decrease the certainty of the evidence					The effect per 1.000 patients tested	Test accuracy CoE
			Risk of bias	Indirect evidence	Inconsistency	Imprecision	Publication bias		
True positives (patients with [condition of interest])	6 studies 95 patients	Cross-sectional (cohort type accuracy study)	Serious	Not serious	Not serious	Serious	Strong association	0 to 0	⨁⨁⨁◯ Moderate
False negatives (patients incorrectly classified as not having [the condition of interest])								0 to 0	
True negatives (patients without [the condition of interest])	1 study 7 patients	Cross-sectional (cohort type accuracy study)	Serious	Not serious	Serious	Not serious	All potential confounding factors would reduce the demonstrated effect	0 to 0	⨁⨁⨁◯ Moderate
False positives (patients with [the condition of interest] incorrectly classified)								1000 to 1000	

As shown in Table 7, of the seven articles analyzed in this SR, 6 had a cross-sectional study design (cohort type accuracy study) with a total N of 95 patients and presented quality of evidence by evaluating the moderate grade system. Despite having a cross-sectional study design (cohort type accuracy study), one of the articles did not evaluate its seven patients with the same criteria used by the other authors. However, it presented similar results to the other studies with the quality of evidence through the GRADE Moderate System's evaluation.

## Discussion

The JBI's systematic review starts with an evidence-based health model that focuses on the best information available and is not exclusively concerned with effectiveness. The model is adaptable to the diverse origins of health problems and uses various research methodologies to generate evidence related to the subject. JBI believes that healthcare professionals need evidence to support a comprehensive range of activities and interventions and, when making clinical decisions, should consider whether their approach is feasible, appropriate, meaningful, and effective. The instruments used to assess bias were critical assessment checklists validated by the JBI for each study design: cohort studies and studies reporting prevalence data and case reports. Four articles^11^, ^20^, ^21^, ^22^ showed a low risk of bias, and three article^19^, ^23^, ^24^ showed a moderate risk of bias. This information corroborates the signaling of the satisfactory quality of the studies found.

This SR showed that not all individuals with ASD and associated hearing loss who underwent cochlear implant developed oral communication. However, the intervention demonstrated other benefits such as increasing interaction with family members, establishing eye contact more frequently, and identifying sounds. The results corroborate previous studies’ findings that children’s gains were small compared to the general population receiving CI; however, the children showed development progress compared to pre- and post-surgery assessments.^19^

Oral communication is not a realistic meta-test in children with ASD and cochlear implants. Nonetheless, the children gained a range of varying functional benefits that traditional methods evaluating the results of cochlear implants in children with autism are generally insufficient to fully assess.^24^ Therefore, future studies evaluating the impact of CI among ASD children need to expand the success criteria assessing the individual’s full development abilities instead of narrowed criteria focused only on acquiring oral communication. This new approach will improve CI intervention recommendations, considering the children’s well-being and quality of life.

Studies argue that objective documentation of performance changes can be difficult or impossible for some children with ASD. In these cases, the authors argued that subjective reports from parents and professionals indicating that the implant had a positive effect on the child and the family might be the only measure of success that can be trusted.^20^ In this RS, 21 patients – 31.8% did not establish any form of traditional communication. However, there was an increase in interaction with family members, this condition being more present in cases of ASD and hearing loss associated with other disabilities. These data exemplify that social interaction development may go unnoticed in traditional assessments but be present in the reports of family members, caregivers, and professionals who monitor these children’s development. This information reinforces the possibility that patients with ASD and hearing loss undergoing cochlear implantation expand other communication skills, even if they do not develop oral communication.

Forty-one (62%) of the individuals participating in the selected articles were diagnosed with ASD after the CI. The CI's mean age was 2.9 years for individuals without other changes and 3.76 years for those with other associated disabilities. It was impossible to determine the mean age for ASD diagnosis, as most studies did not present this information.

There is a worldwide effort to increase the early diagnosis of sensorineural hearing loss and CI intervention at an increasingly younger age. In Brazil, neonatal hearing screening coverage shows an increase from 9.3% to 37.2% in 2008–2015. Although there has been a significant increase in neonatal hearing screening coverage in Brazil in recent years, the national coverage rate is still low (37.2%) and much lower than the recommended literature. Also, in Brazil, there is interregional inequality in the coverage rates of neonatal hearing screening. The South and Southeast regions concentrate as the best rates, while the North, Northeast, and Midwest regions need more efforts to implement the neonatal hearing screening programs.^27^

The diagnosis of ASD is usually made later, considering the process of exclusion diagnosis.^11^ Previous studies have observed that the average time between implantation and the diagnosis of autism was 19 months for most participants and approximately two years after the CI.^21^, ^23^ At the present study, 31 individuals (46.3%) had some other associated disability, such as: cortical blindness, globally delayed, developmental delay, intellectual disability, cerebral palsy, epilepsy, left hemiparesis, visual diffs, prematurity, encephalopathy, gross motor delay, polymicrogyria, gliosis, developmental delay. Similar findings were reported previously, indicating that ASD may be part of a larger picture of multiple disability.^11^, ^20^, ^21^, ^23^ The development of language and oral communication may not be a realistic goal for this audience. There is a great chance that other deficiencies associated with these conditions exist that can aggravate language and oral communication development. Research is needed to search for instruments that can more sensibly measure the development of people with ASD and deafness who have received CI.

Considering the quality of the evidence analyzed by the GRADE System, the results suggest that the CI favors the expressive and receptive language of people with ASD with severe conditions and/or hearing loss profound, even if they do not develop language to the same extent as people who use CI without ASD. Despite the fact that one of the studies does not follow the same line of results as the others selected. Thus, there is a recommendation for CI patients with ASD with associated severe and/or profound hearing loss.

### Strengths and limitations

The limitations of this SR were the reduced availability of articles that met all the inclusion and exclusion criteria of the research question and the diversity of methodologies, measures and evaluation criteria used in the articles found. These facts made it impossible to elaborate the meta-analysis due to the lack of standardization.

As a strategy to overcome these limitations, a detailed description of the study subjects was presented, allowing a qualified discussion of the data with the researched literature. The lack of uniformity in the subjects' performance evaluation tests after the CI and the diversity in the presentation of the results made it difficult to understand the evolution of communication, social interaction in a satisfactory way and the restricted, repetitive, and stereotyped behavior patterns.

## Conclusion

This SR showed that the CI can favor auditory development, language, and social interaction in people with autistic spectrum disorder with associated severe and/or profound hearing loss.

As a strategy to overcome the limitations found in the elaboration of this SR and advance in the area, future studies should look for ways to assess the qualitative development of communication in subjects with ASD and hearing loss submitted to CI. Research protocols must consider the perception of family members, caregivers and professionals who monitor the development of these children. However, protocols must be standardized to allow comparison of results in different samples.

## Conflicts of interest

The authors declare no conflicts of interest.
